# A Randomised Trial of an Eight-Week, Once Weekly Primaquine Regimen to Prevent Relapse of *Plasmodium vivax* in Northwest Frontier Province, Pakistan

**DOI:** 10.1371/journal.pone.0002861

**Published:** 2008-08-06

**Authors:** Toby Leslie, Ismail Mayan, Nasir Mohammed, Panna Erasmus, Jan Kolaczinski, Christopher J. M. Whitty, Mark Rowland

**Affiliations:** 1 Department of Infectious and Tropical Diseases, London School of Hygiene and Tropical Medicine, London, United Kingdom; 2 HealthNet-TPO, Kabul, Afghanistan; Mahidol University, Thailand

## Abstract

**Background:**

Vivax malaria remains a major cause of morbidity in the subtropics. To undermine the stability of the disease, drugs are required that prevent relapse and provide reservoir reduction. A 14-day course of primaquine (PQ) is effective but cannot safely be used in routine practice because of its interaction with glucose-6-phosphate dehydrogenase (G6PD) deficiency for which testing is seldom available. Safe and effective use of PQ without the need for G6PD testing would be ideal. The efficacy and safety of an 8-week, once weekly PQ regimen was compared with current standard treatment (chloroquine alone) and a 14-day PQ regimen.

**Methods and Principal Findings:**

200 microscopically confirmed *Plasmodium vivax* patients were randomly assigned to either once weekly 8-week PQ (0.75mg/kg/week), once weekly 8-week placebo, or 14-day PQ (0.5mg/kg/day) in North West Frontier Province, Pakistan. All patients were treated with a standard chloroquine dose and tested for G6PD deficiency. Deficient patients were assigned to the 8-week PQ group. Failure was defined as any subsequent episode of vivax malaria over 11 months of observation. There were 22/71 (31.0%) failures in the placebo group and 1/55 (1.8%) and 4/75 (5.1%) failures in the 14-day and 8-week PQ groups, respectively. Adjusted odds ratios were: for 8-week PQ vs. placebo-0.05 (95%CI: 0.01-0.2, p<0.001) and for 14-day PQ vs. placebo-0.01 (95%CI: 0.002-0.1, p<0.001). Restricted analysis allowing for a post-treatment prophylactic effect confirmed that the 8-week regimen was superior to current treatment. Only one G6PD deficient patient presented. There were no serious adverse events.

**Conclusions:**

A practical radical treatment for vivax malaria is essential for control and elimination of the disease. The 8-week PQ course is more effective at preventing relapse than current treatment with chloroquine alone. Widespread use of the 8-week regimen could make an important contribution to reservoir reduction or regional elimination where G6PD testing is not available.

**Trial Registration:**

ClinicalTrials.gov NCT00158587

## Introduction

*Plasmodium vivax* is a common cause of malaria in the subtropics. Estimates put the global burden at 70–80 million cases per year [1]. Outside Africa, the disease accounts for more than 50% of all malaria cases, and in Asia it is the major cause of malaria morbidity [2]. Although *P. vivax* causes relatively few deaths there is increasing evidence suggesting that severe and life-threatening complications are more common than previously thought [3]. *P. vivax* has major deleterious effects on development and economic performance both at individual and national levels [4]; those in endemic areas may have 10–30 episodes of vivax malaria in the course of childhood or working life each resulting in 5–15 days absence from work or school.

Conventional transmission control methods targeting the mosquito vector are imperfect owing to the infectious reservoir; dormant hypnozoites in the liver produce episodes of relapse for several years after initial infection [5]. Relapses from hypnozoites coincide with the seasonal abundance of the vector and it is this pattern of relapse which gives the disease its stability in subtropical areas where transmission by mosquitoes is seasonal [6]. Most vivax cases experience relapse and each initial infection typically causes 5–6 subsequent episodes if radical treatment is not administered. Hence a significant proportion of the burden of vivax malaria can be attributed to relapses rather than to infections resulting from transmission [7]. Despite this, there is currently no widely available safe and effective radical cure. In most malaria endemic areas glucose-6-phosphate dehydrogenase (G6PD) deficiency, a heritable enzyme deficiency, is common. For example, in Pakistan and Afghanistan, where *P.vivax* is the predominant species [8], the prevalence of G6PD deficiency varies between ethnic groups and is estimated at 2–10% in the general population [9], [10]. Administration of a 14-day course of primaquine (PQ) (the only recommended regimen that can eliminate the hypnozoite reservoir) to G6PD deficient individuals is contraindicated due to the risk of haemolysis [11].

A truncated 5-day course of PQ for vivax malaria is used commonly in South Asia to reduce the risk of haemolysis and enhance adherence to treatment [12]. Evidence from Pakistan and India demonstrates that the 5-day PQ course is ineffective at reducing relapse rates [13], [14]. One recent review concludes that 5-day PQ is no better than chloroquine alone at radical cure [15]. A study comparing relapse rates in treatment groups that were supervised and unsupervised when treated with 14-day PQ indicated that relapse prevention was similar between the two groups suggesting comparable treatment adherence [7]. However, use of the 14-day course is only recommended where the G6PD status of the individual is known and where adherence can be assured [11]. It is for this reason that the drug is not more widely available [9] since G6PD testing of cases is impractical in most low-resource settings owing to lack of funds, equipment and/or expertise.

Studies from the 1960s provide evidence suggesting that successful PQ therapy is not a function of the length the treatment course, nor of the concentration of drug, but of the total dosage administered. Alving et al [16] administered PQ over 7 days, 14 days, and 8 weeks to African Americans. Each regimen equally prevented relapse in experimentally infected vivax malaria. Extending the course length seems to reduce the risk of haemolysis; Brewer and Zarafonetis [17] showed (in African variant G6PD deficiency) that 8 deficient individuals given 8-week PQ (total dose 410mg) showed no clinical signs of haemolysis. Subjects (n = 8) given PQ twice weekly showed more marked evidence of haemolysis. Further evidence suggests that the haemolytic effects of PQ in G6PD deficient individuals are not produced by the drug itself, but by one or more of its many metabolites [18], [19]. Little is known about the pharmacokinetics and haemolytic potential of the various metabolites [20], [21]. However, by extending the time between PQ challenges clearance of haemotoxic agents (drug metabolites) will be more nearly complete than in shorter courses involving repeated daily dosing. The haemolysis seen in shorter courses (5–7 days) is often self-limiting and there is some evidence that erythropiosis feedback mechanisms up-regulate production of red cells in response to haemolytic challenge once acute malaria is treated [22]. Older erythrocytes are more susceptible to oxidative stress precipitated by PQ challenge and, in concert with the infection, results in a younger and more robust circulating population. The extended course could be safer in G6PD deficient individuals as a result of these inherent safeguards.

The aim of the study was to test whether an 8-week PQ regimen is effective at radical cure without the associated risk of haemolysis in G6PD deficient individuals. This regimen may be appropriate for deployment in resource-poor settings where G6PD testing is unavailable. It was compared to current standard treatment (chloroquine) given with 8-week placebo and to a regimen known to be effective (chloroquine plus 14-day PQ). Since treatment with chloroquine alone is current standard treatment, superiority of the 8-week course over placebo was the primary comparison.

## Methods

### Location

The study was conducted in Adizai, Baghicha and Khagan villages, close to Peshawar, Northwest Frontier Province, Pakistan where Afghan refugees have been resident for more than 20 years. Malaria transmission is seasonal and predominantly due to *P. vivax* (85–95% of cases) [6]. The villages contain Basic Health Units (BHU) run by NGOs, providing free primary health care services to the populations of the villages. Malaria control in the villages was supported by a vertical control program implemented by the NGO HealthNet-TPO and funded by UNHCR. Amongst other services, the control program provides free quality assured microscopy and treatment services for all residents. Local vivax malaria treatment policy is to treat with chloroquine alone while falciparum malaria (now constituting less than 5% of annual cases) is treated with sulfadoxine-pyrimethamine and artesunate.

Ethical approval for the study was granted by the Pakistan Medical Research Council Committee on Bioethics and the London School of Hygiene and Tropical Medicine ethics committee. Permission was also obtained from local government agencies and the United Nations High Commissioner for Refugees. The study was prospectively registered at clinicaltrials.gov (number: NCT00158587). Sponsors and funding agencies (UNDP/World Bank/WHO Special Program for Research in Tropical Diseases; Gates Malaria Partnership) had no role in study design; collection, analysis, and interpretation of data; writing of the paper; or decision to submit for publication.

### Patient Enrolment and Follow-up

Patients attending the basic health units with symptoms compatible with malaria had Giemsa stained thick and thin blood films obtained by finger prick and examined by trained microscopists. Those diagnosed with *P. vivax* infection were asked to participate in the study following informed consent and if they met the inclusion and exclusion criteria. Consent was obtained in writing by patients or their guardians, and in the case of those unable to read was witnessed by a literate person. Inclusion criteria were: Patients diagnosed with *P. vivax* parasitaemia at study BHUs; Patients over 3 years of age; Patient permanently resident in the village. Exclusion criteria were: pregnancy or lactation; severe clinical anaemia (<7g/dl); *P. falciparum* and/or *P. vivax* (mixed infections); intake of any antimalarial drug in the 2 weeks prior to consultation; patients unavailable for the duration of follow up (11 months); patients with concomitant infections or disease likely to mask treatment response.

The study was designed as an open label, randomised, placebo controlled study. Patients were randomly allocated to one of the three treatment groups by study staff in the clinic once informed consent was received and inclusion/exclusion criteria assessed. For practical reasons, two randomisation methods were used. In Baghicha and Khagan villages, patients were randomised by household, whereas in Adizai randomisation was at the individual level. Randomisation lists for each village were generated using a random number list (MS Excel, Microsoft Corp., Seattle, USA) by staff not involved in patient recruitment. Patients were randomised on enrolment by study staff in the BHUs based on house number or sequential patient numbers, depending on the study site.

All patients were treated with initial 3-day chloroquine (25 mg/kg, in divided doses over 3 days) for acute disease. This was accompanied by either supervised weekly placebo once per week for 8 weeks; supervised PQ treatment, daily for 14 days (0.5mg/kg per day); or supervised PQ once per week for 8 weeks (0.75mg/kg per week). All patients were tested for G6PD deficiency at enrolment using a colorimetric test (Sigma Diagnostics, Poole, UK). Those with G6PD deficiency were not randomised, but assigned to the 8-week PQ group in order to follow closely to assess safety.

Patients received directly observed treatment according to the dosing schedule for each treatment group. Each patient was given an appointment card and if the patient was not present on the morning of their scheduled dose they were visited in the afternoon at their home. If the patient was not present they were treated on the next day. If they were absent on this second day, they were counted as losses to follow-up. Each dose was recorded with a signature or finger print of the patient. Patients were monitored during the eight weeks of treatment and for nine additional months by active surveillance (being visited in their homes every two weeks) and passively on presentation at the basic health centre. Patients presenting at the basic health unit with suspected treatment failure (febrile illness) were assessed by thick and thin blood smear. If positive they were classified as a treatment failure and re-treated with the original treatment given at enrolment. Blood slides were double read by two independent microscopists, blinded to the others result.

### Statistical Issues

The primary outcome was the occurrence of any episode of microscopically confirmed vivax malaria over the 11 month observation period which classified the patient as a treatment failure. Sample size estimation was based on the assumption of 32% and 49% failure in treated (14-day PQ) and untreated individuals, as recorded previously in other studies over a 9 month post treatment follow-up [7], [13]. Allowing for 15% loss to follow-up a sample size of 212 per treatment arm was required to detect this difference with 90% power at the 95% confidence level. The incidence of malaria was significantly lower than that seen in previous years leading to lower-than-expected enrolment rates. This led to a revision of sample size following an unscheduled interim analysis conducted in June/July 2006 with the aim of determining whether to halt the trial for futility as it would be unable to hit initial sample size targets because of the low malaria incidence. It was based on data from 88 patients enrolled in Khagan and Baghicha camps who had completed the 11 month observation period. The decision was made that the public health importance of being able to detect a larger difference than that initially calculated remained high, and that the trial should be completed aiming to detect the larger difference of 5% failure in the 8 week PQ group and 30% in the placebo group (ie unable to exclude a smaller difference). A revised sample size of 66 per treatment arm (allowing 10% loss to follow-up) gave a power of 90% at the 95% confidence level to detect this difference. The study was not powered to show equivalence between the 14-day and 8-week PQ arms.

Analysis was conducted on an intention-to-treat basis. In addition to the primary outcome, secondary outcome variables included the number of subsequent episodes and anaemia rates during and up to 2 weeks post-treatment as well as any notable adverse events. Univariate logistic regression analysis using the primary outcome provided crude odds ratios (OR). Potential confounders (sex, age-group and village) were identified on an *a priori* basis and were included in multivariate analysis. Each treatment group was compared in turn to assess superiority (or otherwise) using logistic regression analysis, adjusting for potential confounders. Kaplan-Meier survival analysis using time to first relapse as the endpoint was used to calculate cumulative probability of treatment failure (i.e. having no further episode of malaria). Losses to follow-up were treated as censured data in the analysis. Data was recorded by trained health workers on patient record forms, double entered using Excel XP (Microsoft Corp, Seattle, USA) and analysed using STATA v10.0 (Stata Corp, College Station, TX, USA).

The protocol for this trial and supporting CONSORT checklist are available as supporting information; see Checklist S1 and Protocol S1.

## Results

**Figure 1:**
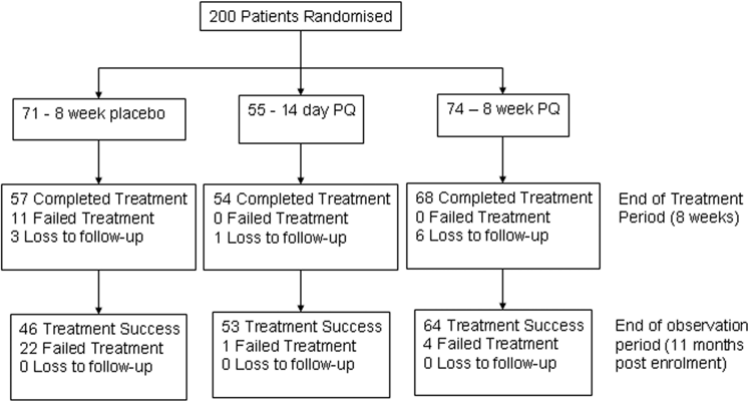
Patient flow through the trial at enrolment, end of treatment period (8 weeks) and end of follow-up (11 months).

200 patients were recruited from 13^th^ September 2004 until 17^th^ July 2006. Follow-up was completed on 16^th^ June 2007. The number (%) recruited into the study at each site was: Adizai 100 (50.0%), Baghicha 79 (39.5%), and Khagan 21 (10.5%). Ten (5.0%) were either lost to follow-up (9) or withdrawn owing to protocol violation (1). Seventy one (35.5%) patients were enrolled to 8-week placebo, 55 (27.5%) to 14-day PQ and 74 to 8-week placebo (37.0%). Figure 1 shows patient through-put during the observation period and Table 1 shows enrolment characteristics.

**Table 1:** Enrolment characteristics of study sample, by treatment group.

	8-week Placebo	14-day PQ	8-week PQ
***Number***	71	55	74
***Number lost to follow-up***	3 (4.2%)	1 (1.8%)	6 (8.1%)
***Study Site***			
*** Adizai***	27	30	43
*** Baghicha***	33	23	23
*** Khagan***	11	2	8
***% Male***	58.6	43.6	48.0
***Median Age, yrs [Range]***	9 [4–50]	10 [4–45]	10 [4–80]
***Median Weight, Kg [Range]***	25 [12–67]	24 [8–70]	29 [11–66]
***Mean Hb on Day 0, g/dl [SD]***	12.0 [1.7]	12.6 [1.9]	12.6 [1.8]
***N (%) anemic (Hb<10.0)***	10 (14.1)	2 (3.6)	7 (9.5)

There were 22/71 (31.0%) failures in the placebo group and 1/55 (1.8%) and 4/75 (5.1%) failures in the 14-day and 8-week PQ groups respectively, giving rates of failure (per 1000/pers months) of 37.3 (95%CI: 24.6–56.6), 1.7 (0.2–12.0) and 5.5 (2.1–14.7), respectively. Treatment group and village were independently associated with treatment failure, with highest failure rates recorded in the placebo group and in Adizai village (Table 2). Sex was not associated with treatment failure. Multivariate logistic regression analysis of treatment outcome, adjusting for sex, age-group and village was conducted to compare each group in turn (Table 3). By this analysis the 8-week PQ regimen was superior to placebo (AOR 0.05 [95%CI–0.01-0.2], p<0.001), as was the 14-day PQ regimen (AOR 0.01 [95%CI–0.002-0.1], p<0.001). Cure rates for the 14 day PQ appear slightly higher than for 8 week PQ regimens (AOR 3.8 [95%CI–0.4-36.7] p = 0.3), however the study was insufficiently powered to demonstrate equivalence or superiority.

**Table 2:** Number (%) with treatment failure in each treatment group over the 11 month observation period, by sex, age group and village.

	8-week Placebo	14-day PQ	8-week PQ
***All***	22/71 (31.0)	1/55 (1.8)	4/74 (5.1)
***Male***	10/41 (24.4)	0/24	2/35 (5.7)
***Female***	12/29 (41.4)	1/31 (3.2)	2/38 (5.3)
***Age Group***			
*** 3–10***	15/46 (32.6)	1/30 (3.3)	4/38 (10.5)
*** 11–20***	7/20 (35.0)	0/19	0/23
*** >20***	0/5	0/6	0/13
***Village***			
*** Adizai***	18/27 (66.7)	1/30 (3.3)	4/43 (9.3)
*** Baghicha***	4/33 (12.1)	0/23	0/23
*** Khagan***	0/11	0/2	0/8

**Table 3:** Univariate and multivariate logistic regression analysis for treatment failure comparing each group. 95% confidence intervals in parenthesis.

	Full study period (11 months observation)		Restricted to failures occurring from 2–11 months^2^		Restricted to failures occurring from the end of treatment+30 days^3^	
	OR	AOR^1^	OR	AOR^1^	OR	AOR^1^
***Placebo vs. 8-week PQ***	0.1 (0.04–0.4)^4^	0.05 (0.01–0.2) 4	0.3 (0.08–0.9)^5^	0.1 (0.03–0.5) 4	0.4 (0.2–0.7) 4	0.3 (0.1–0.5) 4
***Placebo vs. 14-day PQ***	0.04 (0.005–0.3) 4	0.01 (0.002–0.1) 4	0.08 (0.01–0.6) 4	0.03 (0.003–0.3) 4	0.05 (0.006–0.4) 4	0.02 (0.002–0.1) 4
***14-day PQ vs. 8-week PQ***	3.1 (0.3–28.4)	3.8 (0.4–36.7)	3.3(0.4–30.5)	4.2 (0.4–41.3)	3.3 (0.4–30.5)	4.2 (0.4–41.3)

1 Adjusted odds ratios (AOR) adjust for refugee village, sex and age.

2 Restricted analysis excludes all failures during treatment period (months 0–1).

3 Restricted analysis excludes all failures occurring before 33 days for placebo group, 44 days for 14 day PQ group and 91 days for 8 week group. Time under observation is censured at 244 days post restriction period to give equal follow-up period.

4 p<0.001

5 p<0.01

Since a prophylactic effect of PQ administered over the 8 week treatment period may confound true failures, two restricted analyses were conducted which included only those who had failed treatment in the post treatment period. The first was restricted to failures occuring in months 2–11. The second took account of the time the drug was potentially providing a prophylactic effect, and included the period of administration of active drugs plus an additional 30 day period, post-treatment. For this analysis data were censured at 33 days for the placebo group, 44 days for the 14 day PQ group and 91 days for the 8 week PQ group (Table 3). The data was also censured at 244 days post restriction period to give equal periods of observation. During the 2–11 month period there were 11/60 (18.3%) failures in the 8-week placebo group; 1/55 (1.8%) in the 14-day PQ group; and 4/74 (5.4%) failures in the 8-week PQ group. When compared by treatment group and adjusted for age, sex and refugee village, both 8-week and 14-day PQ were superior to 8-week placebo (AOR 0.1, 95% CI: 0.03-0.5, p<0.0001) and (AOR 0.03, 95%CI: 0.003-0.3, p<0.0001), respectively (table 3). After treatment plus 30 days, over the 244 day observation period, results differed marginally (table 3).

**Figure 2:**
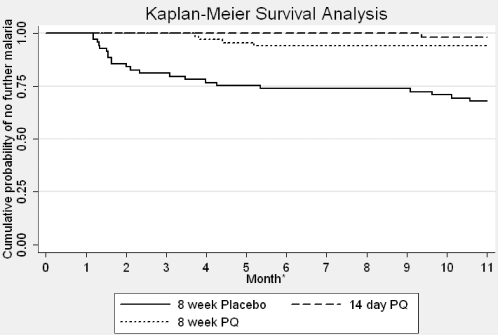
Kaplan Meier Survival Analysis, by treatment group, over 11 months of observation.

**Figure 3:**
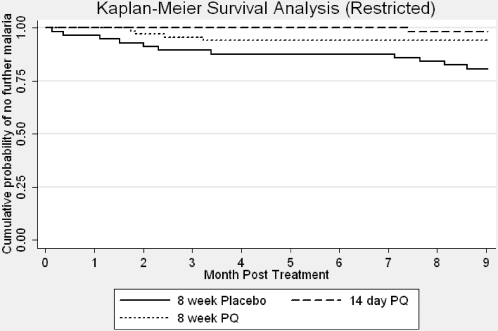
Kaplan Meier survival analysis, by treatment group, restricted to post-treatment period (months 2–11).

Table 4 shows the number of episodes of malaria recorded during the observation period. In the placebo group, 5 additional episodes of malaria were recorded in one patient, whereas only single episodes were recorded in any of the PQ treated patients. Median time to first episode was, 63 days (range 36–322 days) (n = 22) in the placebo group; 285 days (n = 1) in the 14-day group and 125 days (range 113–158) (n = 4) in the 8-week group. There were too few failures for reliable statistical assessment of differences in the median time to failure. Table 5 shows the frequency of first relapse during the 2 month period of treatment and then at 3 month intervals during the follow-up period. In the 8-week placebo group 11/22 (50.0%) of failures were recorded during the first two months. Cumulative probability of treatment failure for each group over the full 11 month observation period was 35.2% (95%CI: 25.3–47.5%) in the placebo group, 3.6% (95%CI: 0.9–13.8%) in the 14-day and 13.5% (95%CI: 7.5–23.7%) in the 8-week PQ group (long-rank test for equality of survivor functions-Chi^2^ = 22.1, p<0.001) (Figure 2). Figure 3 shows the cumulative probability of treatment failure for the restricted analysis, which excluded all failures occurring during the period of treatment (0–8 weeks).

**Table 4:** Frequency of subsequent episodes of malaria in each treatment group over 11 month observation period, number (%).

*Number of Subsequent Malaria Episodes*	8-week Placebo	14-day PQ	8-week PQ
***0***	46 (67.7)	53 (98.2)	67 (93.1)
***1***	12 (17.7)	1 (1.9)	4 (5.6)
***2***	6 (8.8)	0	0
***3***	3 (4.4)	0	0
***4***	0	0	0
***5***	1 (1.5)	0	0

**Table 5:** Number (%) with treatment failure during different stages of follow-up over the 11 month observation period.

	0–2 months* a	3–5 months^b^	6–8 months^ns^	9–11 months^ns^
***8-week Placebo***	11/71 (15.5)	6/60 (10.0)	1/54 (1.9)	4/53 (7.6)
***14-day PQ***	0/55	0/55	0/55	1/55 (1.8)
***8-week PQ***	0/74	3/74 (4.1)	1/71 (1.4)	0/70

* During 8-week treatment period;

a Chi^2^ = 18.3, df = 2, p<0.001;

b Chi^2^ = 5.8, df = 2 p = 0.054;

ns Not significant.

There were no reported serious or notable non-serious adverse events and all treatments were well tolerated. Only 1 G6PD deficient patient, a 13 year-old male, was detected during this trial. On days 7 and 14 (prior to the second and third PQ doses), Hb in this individual fell below the confidence limits of the mean Hb in G6PD normal females aged 12–14 (n = 13). On day 7 Hb was 10.0g/dl vs 12.6g/dl (95%CI: 11.8–13.4) and on day 14 Hb was 10.6 vs 12.6 (95%CI: 12.0–13.3). By day 21 (prior to the fourth dose) Hb in the patient was within the limits of age and sex matched G6PD normal individuals (hb = 12.7 vs 12.6 [95%CI: 11.8–13.5]). The haemoglobin profile of the three treatment arms in the whole sample did not differ. No patient became seriously anaemic (Hb<7.0 g/dl), and no observed anaemia was clinically significant.

## Discussion

The 8-week PQ regimen in combination with chloroquine is effective at curing acute vivax malaria and preventing relapses. Over a period of 11 months, new episodes of vivax malaria were more frequent in the group administered with placebo than in either PQ treated group and the superiority of 8-week PQ over chloroquine alone is demonstrated. The analysis provides information on the effect of an initial PQ treatment as well as repeated doses. The use of incidence of first relapse as the primary outcome provides evidence that the 8-week course is superior to placebo in prevention of episodes of relapse when given as a single course. Patients who presented with a second (or further) episodes were treated with the same regimen as at enrolment. Incidence of recurrent episodes was lower in both the PQ groups; only one recurrent episode was seen in any patient, whereas up to five recurrent episodes were seen in one patient in the placebo group.

The restricted analysis, which used two approaches to exclude failures during the treatment period (when any prophylactic effect would be seen), confirms that both PQ treated groups are superior to placebo in preventing recurrent episodes. The pattern of failure in the 8-week PQ group, where failures occurred between two and three months post treatment, provides possible evidence of a prophylactic effect; the timing and pattern of the failures matches that of the placebo arm once the drug (and metabolites) have cleared. It could be, therefore, that failures (relapses) are simply delayed by a prophylactic effect of 8-week PQ therapy. If this were the case, however, failed patients treated with PQ for an additional eight weeks would be expected to have differing failure rates when censured at different time-points to account for the prophylactic effect. This effect is not seen; none of the 8-week PQ group had second relapse episodes (table 4). The periodicity of initial failures in the placebo group also support this; without radical treatment with PQ, failures occur in two distinct periods–between 0–3 months post treatment and between 6–9 months post treatment (table 5 and figures 2 and 3), where this is not seen in the 8-week PQ group. The 9 month follow up of each patient suggests that both PQ regimens eliminated the hypnozoite reservoir. Even though some PQ metabolites have long half-lives [18], 9 months should be sufficient to allow for clearance of the drug and it's metabolites. This makes it unlikely that the drugs are simply suppressing emergence of merozoites from latent hypnozoites and likely that the 8-week PQ course is eliminating hypnozoites and providing radical cure.

A recent meta-analysis of 14-day PQ trials describes regional variation in PQ efficacy in India, Brazil and Thailand [23]. In untreated patients, relapse rates up to 80% were noted in Thailand. The median relapse rate in treated groups in India was ∼10% (range 7%–21%) whereas in untreated groups it was somewhat higher, at 37%. Relapse rates in all treated groups (in all three countries) was reduced by 14-day PQ therapy. This analysis did not account for background transmission (causing re-infection), which may account for some of the variation seen between regions. It is not possible to distinguish those episodes that are true relapses from those that are re-infections; there is no reliable way of differentiating between the two [24]. Two comparable studies in this region (and, indeed, in these same villages) have shown failures in 14-day PQ groups at 32% and 19% conducted in 1996 and 2000–1, respectively, using 9 month observation periods [7], [13]. However, the villages where the present study was conduced had markedly lower transmission rates at the time; the incidence of vivax cases per annum was <1% in Baghicha/Kagan and 3.3% in Adizai during 2004 to 2007, a 75% reduction compared to 2000–1 when the last study [7] was conducted. Almost all of the cases detected in the population of the villages were enrolled, and thus around 50–60% of patients received anti-relapse therapy. Against the backdrop of very low transmission in this study, the differences seen compared to the earlier trials conducted in the same villages are probably attributable to a marked reduction in the number of transmission (re-infection) cases rather than to changes in drug sensitivity. The low transmission during the study period provides circumstantial evidence for PQ susceptibility in this region. PQ resistant vivax has been reported elsewhere in Asia [25] where it is thought to have developed through the exposure of vivax to the drug when it was widely employed to reduce falciparum gametocytes [26]. In Pakistan and Afghanistan, 5-day PQ for vivax radical treatment, as well as PQ for falciparum gametocytes had been used for many years (although both policies were abandoned in the refugee camps in 2001) despite which vivax appears to remain highly sensitive. Vivax also remains highly susceptible to chloroquine in this region [27] in contrast to other areas (e.g. Papua) [28].

Efficacy and safety data on 8-week PQ is scarce, based on small sample sizes with no comparison group [16], [17]. The present study is the first randomised controlled trial evidence for the efficacy of the regimen, and although effective and safe in this sample, conclusions on safety in G6PD deficient patients in general cannot be drawn. The prevalence of G6PD deficiency in our study population was too low to make comparisons. The 5-day PQ course is frequently used in the region yet there are no reports of serious G6PD related adverse events either in the literature or anecdotally following this regimen. The sole G6PD deficient patient showed a slight drop in haemoglobin which was not clinically significant. This may indicate that in this population, G6PD deficiency is less of a risk to treatment with 8 week PQ than currently assumed. If the eight week regimen is to be considered as policy, assessment of the safety profile in G6PD deficient patients will be needed.

An important issue in broadening access to 8-week PQ therapy is adherence to the regimen. Ensuring adherence to a 2 month, once weekly regimen will require proven interventions at the delivery level. Unsupervised 14-day PQ, accompanied by strong health education messages was equally effective to supervised therapy in terms of treatment outcome amongst Afghan refugees in Pakistan [7]. In Thailand, poor adherence rates to the 14 day course is considered to be responsible for poor cure rates, whereas a high dose (60mg per day) 7 day regimen was shown to be efficacious in G6PD normal individuals over 28 days of follow-up [29]. However, this observation period is short, and our study shows that longer periods of follow up are desirable before coming to firm conclusions on radical cure can be made. However, the contrast between adherence in these differing settings indicates that commonly held concerns or assumptions about adherence to treatment are variable and can be modified. A number of studies have shown that adherence to treatment can be enhanced by relatively simple and inexpensive measures such as blister packaging, aides memoir, health education and improved provider knowledge [30]. These interventions may be similarly appropriate for long-course PQ but will require evaluation before they are taken to scale.

Since the idea of malaria elimination (or eradication) has again reached prominence [31], efforts to improve radical treatment of vivax malaria are required. In the absence of easily administered and widely available G6PD testing, the 8-week course of PQ should be further explored as a tool in preventing recurrent episodes of acute malaria and reducing the infectious reservoir. Widespread use of 8 week PQ has the potential to have dramatic effects on the global burden of vivax malaria.

## Supporting Information

Checklist S1 CONSORT Checklist.
(0.06 MB DOC)

Protocol S1 Trial Protocol.
(0.20 MB DOC)
