# Supplementary material for: A Randomised Trial of an Eight-Week, Once Weekly Primaquine Regimen to Prevent Relapse of Plasmodium vivax in Northwest Frontier Province, Pakistan
Source: PLoS One. 2008 Aug 6;3(8):e2861. doi: 10.1371/journal.pone.0002861 (PMC2481394; doi:10.1371/journal.pone.0002861)
Supplement: Protocol S1 — Trial Protocol. (0.20 MB DOC) [file pone.0002861.s002.doc]

# OPERATIONAL RESEARCH STUDY PROTOCOL

# Version 2.1

#
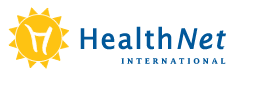


**A multi-arm, placebo controlled, randomised evaluation of an eight-week primaquine regimen using single weekly doses for the treatment of *Plasmodium vivax* malaria**

# © HealthNet International, 2004, all rights reserved.

# Background

­*Plasmodium vivax* is a common cause of malaria in many tropical and subtropical regions. A recent estimate puts the global burden at 70-80 million cases per year^[2]^. Outside Africa *P. vivax* accounts for more than 50% of all malaria cases, and in Asia a high degree of morbidity^[1]^. Despite this there is a relative paucity of research on *P.vivax* with the majority of the literature focusing on the more lethal *P. falciparum*. Although repeated attacks of *P. vivax* cause relatively few deaths they have major deleterious effects on development and economic performance both at individual and national levels; persons in endemic areas may have 10-30 episodes of vivax malaria in the course of childhood or working life each resulting in 5-15 days absence from work or school^[2]^. Further, risk of disease is higher in those who are the major economic and care providers for their families^[2]^. Conventional control methods are inefficient^[3]^, due to the infectious reservoir, in the form of hypnozoites in the liver, producing episodes of relapse for several years after initial infection^[4]^. Each initial infection may result in 5-6 subsequent episodes; therefore a significant proportion of the burden of vivax malaria can be attributed to relapses, and not initial infections^[10]^. In Afghanistan and Pakistan this problem is compounded by inequalities in access to quality health care services (the majority of sufferers are from impoverished communities of refugees and IDPs living on marginalized land) and the low age of disease risk (~2-25 years) resulting in educational impairment, and decreased productivity. The effect of malaria on economic development is now increasingly recognized, with its effect on absenteeism and acquisition of capital having been explored in detail^[12]^.

Despite the fact that a substantial proportion of the burden of vivax malaria is caused by relapse there is currently no safe and effective cure. In Pakistan and Afghanistan, where *P.vivax* is the predominant species^[6]^, glucose 6 phosphate dehydrogenase (G6PD) deficiency is a common heritable trait amongst Pakistani and Afghan Pashtun populations (~10%) ^[7]^. Administration of a 14-day course of primaquine (PQ) (the only regimen which can eliminate the hypnozoite reservoir)^[5]^ to G6PD deficient individuals is contraindicated due to the risk of haemolysis. National governments in Asia have adopted a truncated 5-day course of PQ for vivax malaria as this reduces the risk of haemolysis to negligible levels and increases adherence rates^[5]^. However, Rowland & Durrani^[8]^ have shown that the 5-day PQ course is ineffective at reducing relapse rates amongst Afghan refugees in Pakistan but that a supervised 14-day course is effective in those without G6PD deficiency. This is also confirmed by a study in India^[9]^. A further study comparing relapse rates in treatment groups that were supervised and unsupervised when treated with 14 day primaquine indicated that relapse prevention was comparable between the two groups^[10]^. Use of the 14 day course, therefore, is only recommended where the G6PD status of the individual is known and where adherence can be assured^[5]^. G6PD testing of all suspected cases is impossible in low-resource settings with neither funds nor expertise available; therefore the only option to be explored for those living in poverty is to find novel ways to administer PQ.

Old evidence suggests that successful PQ therapy is not a function of the length of the course of treatment, nor the circulating concentration of the drug, but of the total dosage administered. Alving et al^[11]^ showed that the same dose administered over 7 days, 14 days, and 8 weeks equally prevented relapse in vivax malaria. Further evidence exists suggesting that the haemolytic effects of PQ in G6PD deficient individuals are not produced by the drug itself, but by one or more of its many metabolites (9 have so far been identified)^[13]^. Although much is left to be elucidated regarding the pharmacokinetics and haemolytic potential of the various metabolites, a number of putative haemotoxic agents have been proposed^[13]^. These are likely to have differing pharmacokinetic properties, such as extended half lives and high peak plasma concentrations^[14, 15]^. The logical conclusion, therefore, is that by extending the time between PQ challenges, clearance of hemotoxic agents will be more nearly complete than in a relatively short course. In addition, the hemolysis seen in shorter courses is often self-limiting, suggesting that feedback mechanisms up-regulate production of red cells in response to haemolytic challenge^[16]^. The proposed extended course is likely to be well tolerated in G6PD deficient individuals as a result of these safeguards, especially in this population which has been exposed to the 5 day PQ regimen for a number of years without reported adverse effects.

The aim of the study, therefore, is to test whether an 8 week regimen proves effective, without the associated risk of haemolysis in G6PD deficient individuals. This would be appropriate for deployment in a resource-poor setting if it proved effective and safe.

## Objectives

1. Determine the efficacy of an eight-week primaquine regime, using single weekly doses, in preventing relapses of *P. vivax* infections.
2. Determine the efficacy of the above regime to reduce the overall parasite load in patients suffering from *P. vivax* infections, thereby reducing disease transmission.
3. Monitoring of the effect of 8 week PQ on G6PD deficient individuals.

# Methodology

## *Location*

The study will be conducted in Baghicha and Khagan camps, close to Mardan, with a high burden of *P.vivax*, located in North West Frontier Province (NWFP), Pakistan.

#### Laboratory Examination

Blood will be obtained by finger prick from all symptomatic suspected malaria cases attending the basic health unit (BHU) at Adizai. Thick and thin film films will be prepared, air dried and stained in 10% buffered Giemsa’s stain for 10 minutes, then washed and air dried. Slides will be examined by conventional light microscopy, under oil immersion at X 100. Cases diagnosed with *P. vivax* infection will be asked to participate in the present study unless the exclusion criteria are met.

Patients will be tested for G6PD deficiency by near patient testing (Sigma Diagnostics, UK). This will require 0.05 ml of blood. The test is a colorimetric qualitative test for G6PD in red cells, and will be carried out by a trained laboratory technician on-site in the two BHUs

***Inclusion Criteria***

- Patients diagnosed with *P. vivax* parasitaemia at study BHUs
- Patients over 3 years of age
- Patients with G6PD deficiency to a sub-study, safety trial
- Patients without G6PD deficiency to all other groups.

##### Exclusion criteria

- Children under the age of three
- Pregnant / breast feeding women
- Patients with severe clinical anaemia (<7g/dl)
- Patients with *P. falciparum* or mixed infections
- Patients unavailable for the duration of follow up.
- Patients who have taken any antimalarial drugs in the 2 weeks prior to consultation.
- Patients with concomitant infections or whose general health is considered too poor.

## *Design*

Randomised placebo controlled study comparing the following three study groups for the general population:

1. Initial 3 day chloroquine with supervised weekly placebo for 8 weeks (-ve control group)
2. Initial 3 day chloroquine followed by supervised 14 day primaquine treatment (+ ve control group) – excluding G6PD deficient patients.
3. Initial 3 day chloroquine followed by supervised 8 week primaquine treatment (45 mg / week)

All patients will be given health education messages and strongly advised to complete the course. In addition a safety arm will be used for G6PD deficient patients; this will be used to compare HB with group 3 (above). This will be an 8 week regimen, administered by direct observation, and will receive close monitoring for signs of haemolysis. See annex I.

#### Sample Size

Calculation of the required sample size was based on estimates of previous primaquine studies in Afghan refugee camps. A second episode of *P. vivax* infection (i.e. relapse) has been estimated at 32% and 49% in the supervised therapy and control groups respectively. Based on these assumptions, a sample of 185 individuals is required for each of the three groups, allowing for 90% power of the study to detect the given difference (or greater) at 95% confidence level. Allowing for a 15% loss to follow-up, 212 patients with laboratory diagnosed *P. vivax* will need to be included in each of the four study groups. The sample size may be adjusted during the study depending on preliminary results. A third episode (i.e. second relapse) has been estimated at 3% and 20% in treated vs untreated groups. This will require a smaller sample size (56 in each group) allowing for 90% power and 95% confidence level to detect the given difference.

##### Randomisation

All patients will be randomly allocated into three groups using randomisation blocks of different sizes. In each compound all cases will be given the same treatment (see above).

##### Treatment

All patients included in the study will be given a full therapeutic course of chloroquine. Primaquine will be administered to the study groups according to the respective protocol schedule. All relapses and new cases will be re-treated using the same regime and procedure as was at the time of admission. See Annex III for dosage tables.

### *Timeframe*

The study will start in July 2004. Recruitment will be continued until November 2004 (or until the required number have been recruited, if this is before October). Patients will be followed up for a period of one year after recruitment into the study. Therefore the study will conclude in November 2005.

#### Training

Staff of the BHU and community health workers will receive instruction on the study design and their respective roles in diagnosis, recruitment, laboratory techniques and administration of anti-malaria drugs.

##### Ethical Considerations

All patients fulfilling the entry requirements will be required to give informed consent before being recruited for the study. All patients, including those not willing to participate, will receive standard treatment with chloroquine upon diagnosis (25 mg / kg). All patients in the control group will also receive primaquine at the end of the study, if efficacy is proven.

Whilst it is known that 14 day PQ is an effective radical cure for *P. vivax* in this region the justification for placebo control in this trial is that chloroquine alone is the current best available treatment, and the current treatment policy for these camps. The justification for the use of G6PD deficient individuals in this study is self-evident; the only radical cure currently available in this region, 14 day PQ is contraindicated, and, the absence of affordable G6PD testing at health facilities makes the 8 week regimen, if proven safe in this group of individuals, the only option for a sustainable treatment policy in this region of the world.

Ethical approval has been granted by the Ethics Committee of the London School of Hygiene & Tropical Medicine and from the Pakistan Medical Research Council Committee on Bioethics.

###### Monitoring

All patients will be monitored for the initial 8 weeks of treatment (including the 14 day primaquine group) for signs of haemolysis; G6PD deficient patients will be monitored, by HB, every other day for the first 2 weeks of treatment and then once per week until completion of the course. Following the treatment period, patients will be monitored for 1 year for the following outcomes:

Primary Efficacy Variable: Proportion with subsequent episode of *P. vivax* in 9 months of follow-up. [it is assumed that all trial participants have an equal chance of acquiring new infections and thus differences in proportion with subsequent infection can be attributed to relapse]

Secondary Efficacy Variables: Time to 1^st^ relapse episode

Number of relapse episodes in 9 months of follow-up

Side effects / adverse events (haemolysis, HB at day 0, 3 and after 8 weeks, total RBC)

Patients will be advised to return to the BHU in the event of recurrent symptoms within 9 months following enrolment to the study. A patient will be deemed to have completed the study if they have completed the course of treatment (accept the unsupervised group) and after 9 months of follow-up.

In any event each patient will be contacted once every 2 weeks during their post treatment follow-up period in order to collect information about adverse events, concomitant medication and any symptoms that could be attributed to malaria, or adverse events.

Patients will be strongly advised to contact the BHU (or the BHU coordinator after-hours) in the event of any severe or moderate adverse event at the earliest possible opportunity.

###### Results

Clinic staff and PHC workers will disseminate the results to the Federal/Provincial Directorate of malaria, the refugee communities, UNHCR, the Pakistan Medical Research Council (PMRC) and HNI’s implementing partners. Depending on the study outcome, treatment guidelines may be revised in discussion with UNHCR and PMRC. Study findings will be published.

***Trial Safety***

The monitoring of adverse events is an important aspect of the trial. ALL ADVERSE EVENTS WILL BE REPORTED TO THE TRIAL CLINICIAN AND THE TRIAL SUPERVISOR AT THE EARLIEST OPPORTUNITY. An adverse event is defined as any noxious, pathological or unintended change in anatomical, physiological or metabolic functions as indicated by physical signs, symptoms and/or laboratory changes occurring in any phase of the clinical study whether associated with the study drug or placebo and whether or not considered drug related. Adverse events will be classified as follows:

Mild: For example, an adverse experience which is easily tolerated by the subject, causing minimal discomfort and not interfering with everyday activities, e.g. vomiting.

Moderate: For example, an adverse experience which is sufficiently discomforting to interfere with normal everyday activities e.g. headache.

Severe: For example, an adverse experience which prevents normal everyday activities e.g. haemolysis.

The trial may be prematurely abandoned if adverse events are reported and are so serious that it is deemed necessary by the investigators. Any adverse events that are attributed to the drug regimen will be reported in the final report and any published articles.

***Analysis:***

Analysis will take place at the end of the trial, by intention to treat. Potential confounding factors will be identified by examining the association between each potential confounder, which will be decided on an *a priori* basis, and the outcome; those factors that are associated independently with outcome at the 10% significance level will be adjusted for in the analysis.

**Requirements**

***Staff requirements***

- 1 x Trial supervisor
- 1 x Medical doctor (for patient examination and early diagnosis of possible side effects)
- 5x CHWs (for patient follow-up)
- 1 x Microscopist

***Material requirements***

- G6PD diagnostic kits
- Primaquine and chloroquine
- Laboratory consumables
- Two workshops; one at the start of the trial for staff training and orientation, the other after completion of the study for dissemination of results, to influence policy change if required
- 440 blister packs each containing 8 PQ tablets of 15 mg each.

***Funding requirements***

- Local staff salaries
- G6PD laboratory diagnosis
- Laboratory consumables
- Two workshops; one at the start of the trial for staff training and orientation, the other after completion of the study for dissemination of results, to influence policy change if required
- Blister packaging of drugs.
- Transport (1 rental car)

***Investigators:***

Toby Leslie^1,2^, Jan Kolaczinski^1,2^ , Nasir Mohammed^1^, M Ismail Mayan^1^, Naveeda Rehman^3^, Panna Erasmus^1^,

Chris Whitty^1^, and Mark Rowland^1,2^

^1^ HealthNet International, MLCP, Peshawar, Pakistan,

^2^ London School of Hygiene & Tropical Medicine, London, UK

^3^ UNHCR, Islamabad, Pakistan

***References:***

1.Roll Back Malaria. [www.mosquito.who.int](http://www.mosquito.who.int/). **2003**

2. Mendis, K, et al. The neglected burden of *Plasmodium vivax* malaria. *American Journal of Tropical Medicine & Hygiene*, 64**,**(1-2 suppl) 97-106. **2001**.

3. Rowland, M et al. Malaria epidemiology and control in refugee camps and complex emergencies. *Annals of Tropical Medicine & Parasitology*, 95, 741-754. **2001**.

4. Garnham, P.C.C. Hypnozoites and relapses in *Plasmodium vivax* and in vivax-like malaria. *Tropical & Geographical Medicine*, 40**,** 187-195. **1998**.

5. *Practical Chemotherapy of Malaria*. Geneva: World Health Organisation, Technical Report Series, no.805. **1990**.

6. Rowland M, et al. Anopheline vectors and malaria transmission in eastern Afghanistan. *Transactions of the Royal Society of Tropical Medicine & Hygiene*. 96(6):620-6. **2002**

7. Bouma M.J et al. Prevalence and clinical presentation of glucose-6-phosphate dehydrogenase deficiency in Pakistani Pathan and refugee communities in Pakistan; implications for the use of primaquine in regional malaria control programs. *Transactions of the Royal Society of Tropical Medicine & Hygiene*, 89, 62-64. **1995**.

8. Rowland M. et al.. Randomised controlled trials of 5 and 14 day primaquine therapy against relapses of malaria in an Afghan refugee settlement in Pakistan. *Transactions of the Royal Society of Tropical Medicine & Hygiene*, 93, 642-643. **1999**.

9. Gogtay N.J. et al. Efficacies of 5 and 14 day primaquine regimens in the prevention of relapses in *Plasmodium vivax* infections. *Annals of Tropical Medicine & Parasitology*, 93, 809-812. **1999**.

10. Leslie et al. Compliance with 14-day primaquine therapy for radical cure of *vivax* malaria – a randomized placebo-controlled trial comparing unsupervised with supervised treatment. *Transactions of the Royal Society of Tropical Medicine and Hygiene,* In Press. **2004**.

11. Alving A.S. et al. Mitigation of the haemolytic effect of primaquine and enhancement of its action against exoerythrocytic forms of Chesson strain of *Plasmodium vivax* by intermittent regimens of drug administration. *Bulletin of the World Health Organization*, 22**,** 621-631. **1960**.

12. Sachs J. et al. The economic and social burden of malaria. *Nature*, 415 (6672), 680-685. **2002**.

13. Bolchoz, L.J.C., et al,. Primaquine induced haemolytic anaemia: Formation and hemotoxicity of the arylhydroxylamine metabolite 6-methoxy-8-hydroxylaminoquinoline. *J Pharmacol Exp Ther*. 297(2), 509-515. **2001**

14. Fletcher, K.A., et al.. Studies on the mechanisms of oxidation in the erythrocyte by metabolites of primaquine. *Biochem Pharmacol.* 37(13) 2683-2690. **1988**

15. Mihaly, G.W., et al,. Pharmacokinetics of Primaquine in man. I. Studies of the absolute bioavailability and effects of dose size. *Br. J. Clin. Pharmac.* 19, 745-750. **1985**

16. Clyde, D.F. Clinical problems associated with the use of primaquine as a tissue schizontocidal and gametocytocidal drug. *Bull WHO*. **59**(3), 391-395. **1981.**

***Annex I: Trial Schematic:***

Consent

**G6PD Test**

Randomisation

Vivax patient, presenting at BHU, confirmed by microscopy.

**Group 1**: 3 days CQ, 8 Wk Placebo

-ve Control

**Group 2**: 3 days CQ, 14 day PQ

+ve Control

**Group 3**: 3 days CQ, 8 Wk PQ (supervised)

**Primary:** Odds of relapse in 9 months post treatment

**Secondary:** Time to 1^st^ episode of malaria

**Secondary:** Number of relapses in 9 month follow up.

**Secondary:** Adverse events.

**Intervention**

**Efficacy Variables**

**Recruitment**

**F**

**O**

**L**

**L**

**O**

**W**

**U**

**P**

**9**

**Months**

**Safety Group – G6PD deficient patients only**: 3 days CQ, 8 Wk PQ (Directly observed,)

Exclusion Criteria:

- Pregnancy
- <3yrs
- Severe Anaemia
- Pf or mixed infection
- Other Disease
- Consent / collaboration
- Recent Antimalarial
- G6PD deficiency (from Group 2)

***Annex III: Dosage Charts.***

1. Primaquine:

|  | | |  |  |
| --- | --- | --- | --- | --- |
| Tablets = 15 Mg | |  |  |  |
| Dosages taken from: | |  |  |  |
| Clyde, D. F. (1981) Bull WHO **59**(3): 391-395 | | | |  |
|  |  |  |  |  |
| Dosage: Mg Base (no Tabs) | | |  |  |
|  |  |  |  |  |
|  |  |  |  |  |
|  |  |  | *Age Group* | |
| *Treatment* | *Frequency* | *4-8* | *9-14* | *>15* |
|  |  |  |  |  |
| *14 Day* | *Daily* | 7.5 (0.5) | 15 (1) | 22.5 (1.5) |
|  |  |  |  |  |
| *8 week* | *Weekly* | 15 (1) | 30(2) | 45(3) |
|  |  |  |  |  |
|  |  |  |  |  |

1. Chloroquine:

| *Tablets = 150mg* | |  |  |  |  |  |
| --- | --- | --- | --- | --- | --- | --- |
| Dosages taken from: | |  |  |  |  |  |
| WHO, (2000) The use of antimalarial drugs. WHO/CDS/RBM/2001.33 | | | | | |  |
|  |  |  |  |  |  |  |
| Dosage Mg Base (No Tabs) | | |  |  |  |  |
|  |  |  |  |  |  |  |
|  |  |  |  |  |  |  |
|  |  |  |  | *Age Group* | |  |
| *Treatment* | *Frequency* | *3-4* | *5-7* | *8-10* | *11-13* | *>=14* |
|  |  |  |  |  |  |  |
| *Day 1* | *1* | 150 (1) | 225 (1.5) | 375 (2.5) | 450 (3) | 600 (4) |
|  |  |  |  |  |  |  |
| *Day 2* | *1* | 150 (1) | 225 (1.5) | 375 (2.5) | 450 (3) | 600 (4) |
|  |  |  |  |  |  |  |
| *Day 3* | *1* | 150 (1) | 150 (1) | 150 (1) | 300 (2) | 300 (2) |
